# Detailed Vascular Anatomy of the Human Retina by Projection-Resolved Optical Coherence Tomography Angiography

**DOI:** 10.1038/srep42201

**Published:** 2017-02-10

**Authors:** J. P. Campbell, M. Zhang, T. S. Hwang, S. T. Bailey, D. J. Wilson, Y. Jia, D. Huang

**Affiliations:** 1 Casey Eye Institute, Oregon Health & Science University, 3375 SW Terwilliger Blvd, Portland, OR 97239 USA

## Abstract

Optical coherence tomography angiography (OCTA) is a noninvasive method of 3D imaging of the retinal and choroidal circulations. However, vascular depth discrimination is limited by superficial vessels projecting flow signal artifact onto deeper layers. The projection-resolved (PR) OCTA algorithm improves depth resolution by removing projection artifact while retaining *in-situ* flow signal from real blood vessels in deeper layers. This novel technology allowed us to study the normal retinal vasculature *in vivo* with better depth resolution than previously possible. Our investigation in normal human volunteers revealed the presence of 2 to 4 distinct vascular plexuses in the retina, depending on location relative to the optic disc and fovea. The vascular pattern in these retinal plexuses and interconnecting layers are consistent with previous histologic studies. Based on these data, we propose an improved system of nomenclature and segmentation boundaries for detailed 3-dimensional retinal vascular anatomy by OCTA. This could serve as a basis for future investigation of both normal retinal anatomy, as well as vascular malformations, nonperfusion, and neovascularization.

Our current understanding of the retinal vascular networks developed from pioneering work on primate histology^1^,^2^. From these early studies, we know that there are up to four retinal vascular networks in the macula (Fig. 1)^3^. The superficial vascular plexus (SVP) is supplied by the central retinal artery and composed of larger arteries, arterioles, capillaries, venules, and veins vessels primarily in the ganglion cell layer (GCL). There are two deeper capillary networks above and below the inner nuclear layer (INL) referred to as the “intermediate” and “deep” capillary plexuses, or ICP and DCP, respectively, which are supplied by vertical anastomoses from the SVP^1^,^2^. The fourth network is a regional layer called the radial peripapillary capillary plexus (RPCP). The radial peripapillary capillaries have a unique anatomic organization because they run in parallel with the NFL axons, as opposed to the deeper vascular plexuses, which have a lobular configuration^4^,^5^. The functional significance of the RPCP has been recognized due to its role supplying the densely packed nerve fiber layer (NFL) bundles in this region^4^,^5^,^6^. This organization of the retinal vascular plexuses has been confirmed in humans *ex vivo* using confocal microscopy^7^,^8^ and *in vivo* using speckle variance OCT^9^ and adaptive optics confocal scanning laser ophthalmoscopy (AO-cSLO)^10^ in research studies, technologies with a very narrow field of view and which are not widely available. Current clinical imaging of the ocular circulation has been dominated by fluorescein angiography (FA), a 2D imaging modality that was developed in the 1950’s that captures the fluorescence signal from intravenously injected dye in the retinal and choroidal circulation, and many retinal conditions are defined by their characteristic FA patterns. FA has the ability to visualize breakdown of the blood-retinal barrier (with the finding of “leakage” of extravasated fluorescein), but is limited in its ability to resolve the depth of specific signal abnormalities, and cannot visualize the deeper retinal capillary layers (ICP and DCP) due to blocked fluorescence^11^,^12^.

Optical coherence tomography (OCT) is a non-invasive imaging technology that has the high speed required for 3D volumetric imaging and the high spatial resolution to visualize individual layers in the retina. OCT has become routine in the evaluation of retinal and optic nerve diseases, and is the most commonly used imaging modality in ophthalmology^13^. In a recently developed extension of OCT, OCT angiography (OCTA) detects blood flow down to the capillary level by measuring change (decorrelation or variance) in OCT signal in consecutive cross-sectional images (B-frames) taken at the same location. Because OCTA uses motion as intrinsic contrast, extrinsic contrast such as intravenous injection of fluorescein dye is not needed, making this new modality more practical for routine clinical use. Since its clinical introduction, OCTA has been used for *en face* visualization of blood flow in anatomic slabs, so that the retinal circulation can be displayed separately from the choroidal circulation, and the vascular anatomy can be further subdivided into more superficial and deeper retinal vascular plexuses, typically called the superficial and deep plexuses^14^,^15^. However, these early attempts to separate the retinal circulation into superficial and deep plexuses has been marred by segmentation boundaries that imperfectly segment the intermediate capillary plexus (Fig. 1), and flow projection artifact^16^,^17^,^18^.

Flow projection artifact is caused by the flickering shadow from superficial blood flow being projected onto deeper tissue layers. Since *in-situ* flow and its shadow both produce OCT signal variation, the OCTA algorithms interpret them both as flow. Due to the projection artifact, superficial vessels acquire a long tail downward (also called tailing artifact) on cross-sectional OCTA. On *en face* OCTA, the vascular patterns from more superficial plexuses are duplicated on the deeper layers, making it impossible to obtain a clean image of the deeper vascular plexuses, or to separately visualize the ICP and the DCP, and demonstrating signal in the normally avascular outer retina. This has complicated early attempts at using OCTA to identify pathological neovascularization in (or under) the outer retina^19^, and quantify nonperfusion in the deeper layers^20^.

We previously reported a novel algorithm called “projection-resolved” OCTA (PR-OCTA)^21^. Our hypothesis was that this algorithm would allow visualization of the four human retinal vascular plexuses known from histology, and allow quantitative comparison of vessel densities at each location in the retina, both in depth (within the layers of the retina), and in location between the optic nerve and the retinal periphery. We proposed to resolve the flow projection artifact limiting existing technology, produce clean *en face* OCTA of the deeper retinal capillary plexuses, and describe the inter-plexus anatomy^18^. Using this technology to characterize the retinal vascular plexuses *in vivo*, we propose a standard nomenclature for these vascular networks, and provide guidance for rational segmentation algorithms, which respect the normal distribution of vascular networks in the human retina.

## Results

### Human Subjects

We examined one eye from each of nine human subjects, including 3 women and 6 men, 3 left eyes and 6 right eyes. The mean age was 30.2 (standard deviation [SD] 7.4). The mean refractive error was −1.3 diopters (SD 1.7). 4 of the subjects were Asian, and 5 were Caucasian. The mean systolic blood pressure was 113 mmHg (standard deviation 4), and none of the subjects had any medical or ocular comorbidities, and were on no topical or systemic medications.

### Anatomic localization of vascular plexuses

Using the PR-OCTA algorithm, we were able to detect the depth of vascular plexuses in the retina in four geographic regions temporal to the optic nerve (peripapillary, parafoveal, perifoveal, peripheral) by identifying depth-specific peaks in vessel density for each subject. This montage OCTA (Fig. 2) from one subject displays decorrelation signals from a single 2 dimensional image demonstrating the typical angiographic appearance of the inner retina with both large and small vessels. Overlaying the decorrelation signal onto a structural OCT image (Fig. 2), after application of the PR-OCTA algorithm, the specific depth and relative breadths of the four vascular plexuses are easily visualized.

Depth-resolved capillary density profiles (Fig. 3) were obtained in 4 anatomic areas. In the peripapillary region, the RPCP and the SVP demonstrate two peaks of capillary density, but with no distinct trough separating them. In the parafoveal and perifoveal regions, there are three distinct peaks corresponding to the three vascular plexuses (SVP, ICP, and DCP). There are 2 distinct troughs separating the 3 plexuses in the central macula (Fig. 3), and the 3 plexuses could be visualized as distinct layers in cross-sectional OCTA vessel density map (Fig. 4). The 3 plexuses merge into one at the edge of the foveal avascular zone (FAZ). In the peripheral region, the ICP and the DCP merge into a single peak (Fig. 3). The merged parafoveal capillary ring can be visualized in the *en face* OCTA of all 3 plexuses (Fig. 5). On the cross-sectional capillary density map (Fig. 4), the ICP and DCP can be seen to merge into a single layer approximately 6–7 mm temporal to the fovea.

### En face visualization of vascular plexuses

Using the depth locations of the retinal vascular plexuses defined by the investigation above (Fig. 3), we produced en face OCTA of the four vascular plexuses (Fig. 5). Although the RPCP and the SVP appear to be a single complex in cross section (Fig. 4), they present distinct patterns *en face* (Fig. 5). The RPCP has long radial capillaries that run parallel with the nerve fiber bundles, whereas the SVP consists of a mixture of large and small vessels (Fig. 5). In the macula, the SVP has a pattern of centripetally branching pattern that terminate at capillary ring around the foveal avascular zone (Fig. 5). The largest arcade vessels interweave between the NFL and GCL, and thus are captured in both the RPCP and SVP slabs. The ICP and DCP consist of thin layers of capillaries arranged in lobular patterns without directional preference.

### En face visualization of inter-plexus layers

In the parafoveal and perifoveal retina, the retinal layers are sufficiently thick for PR-OCTA to resolve not only the 3 distinct vascular plexuses, but also the capillary-free interconnecting layers between them. This is demonstrated in Fig. 6 using a high resolution 2 mm × 2 mm scan in the temporal macula. In these inter-plexus spaces, some interconnecting vascular segments can be observed. Comparison with a color fundus photograph showed that these interconnecting vessels arise from terminal arterioles and venules in the SVP. The diving venules are seen more clearly than diving arteriole in the ICP, DCP, and interconnecting layers (Fig. 6). In some instances, these diving venules are seen to arise from a radiating network of capillaries in the ICP and DCP.

### Regional vessel density variation in the retinal plexuses

The densities of the retinal vascular plexuses show characteristic regional variations (Fig. 7, Table 1). The ICP and DCP have roughly constant density in the peripapillary and macular regions (Table 1) except for the focal absence in the fovea (Fig. 7). They merge into a single layer in the periphery with slightly higher combined density than the single plexuses. The SVP density is highest in the peripapillary and macular regions (except for the foveal area) and decreases in the periphery (P < 0.05 peripheral v. peripapillary, parafoveal, or perifoveal density). In the periphery, the density of the merged ICP/DCP plexus is higher than the SVP (P < 0.05). In the peripapillary, parafoveal, and perifoveal locations (Table 1), the peak vessel density of the SVP is higher than the ICP and DCP (P < 0.05). The RPCP density varies dramatically with location. It is densest near the optic disc edge and decreases outward (Fig. 7). It also appears to range further from the disc along the arcuate nerve fiber bundles (Fig. 5), but the limited imaging area in this study do not permit complete characterization of its distribution. The intravisit repeatability of vessel density measurements was 1.9% for the RPCP, 2% for the SVP, 1.7% for ICP, and 3.3% for the DCP. There were no detectable associations of peak vessel density (parameters shown in Table 1) with age, gender, or refractive error in this small population using multivariable linear regression.

## Discussion

**Figure 1:**
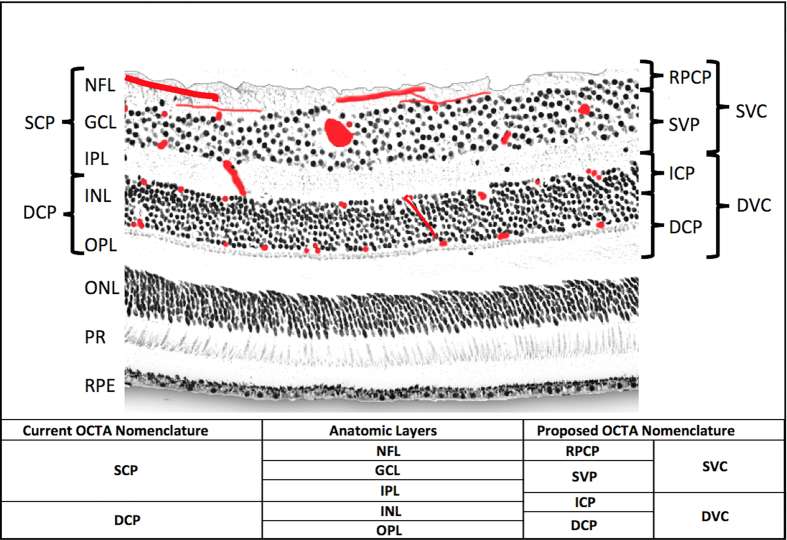
Anatomic localization of vascular plexuses in the human retina in the macula, and current and proposed optical coherence tomography angiography segmentation boundaries. An illustration of the retinal vascular plexuses in red (labeled on right) hand drawn on top of a histological section of the human retina showing anatomic layers (labeled on left) from spectral domain optical coherence tomography. The four vascular plexuses can be grouped into superficial and deep vascular complexes (SVC and DVC, as shown on right) for routine segmentation, but ought to reflect the anatomic location of the ICP at the IPL/INL interface, which the current OCTA segmentations use as a border between superficial and deep plexuses (labeled on left as SCP and DCP). Current and proposed vascular nomenclature and OCTA segmentations are shown at the bottom. (NFL = nerve fiber layer, GCL = ganglion cell layer, IPL = inner plexiform layer, INL = inner nuclear layer, OPL = outer plexiform layer plus Henle’s fiber layer, ONL = outer nuclear layer, PR = photoreceptor layers, RPE = retinal pigment epithelium, OCTA = optical coherence tomography angiography, RPCP = radial peripapillary capillary plexus, SVP = superficial vascular plexus, ICP = intermediate capillary plexus, DCP = deep capillary plexus).

The recent clinical introduction of OCTA enabled 3-dimensional visualization of retinal and choroidal circulations that was not possible with 2-dimensional FA images. Within the retina, the FA image is dominated by the SVP and the choroidal signal, with poor visualization of the ICP and DCP^12^,^22^,^23^. OCTA *en face* images have been used to visualize vascular networks within the retina at various depth layer-by-layer using thin slabs^15^. Using this approach, 2 different vascular patterns were initially distinguished, and they were referred to as the superficial and deep plexuses based on their relative depth and vascular patterns by Lumbroso and others (Figs 1 and 4)^11^,^15^. Spaide *et al*. further noted a radially oriented vascular network could be visualized near the optic disc, corresponding to the radial peripapillary capillaries previously known from histology, but not well visualized by FA^11^. These observations led to many early studies with OCTA in both normal and diseased eyes^24^, however these analyses have been significantly limited due to flow projection artifact^16^,^23^, which prevented: (1) the ability to separate the ICP from DCP, (2) precise visualization of abnormal neovascularization in avascular areas of the retina (as in macular degeneration), (3) accurate quantification of nonperfusion in normally vascularized areas of the retina (as in diabetes), and (4) visualization of the inter-plexus spaces. Indeed, with conventional OCTA all retinal layers, including the avascular outer retina, appeared to contain flow signal, and no natural boundary could be defined between vascular plexuses. Additionally, due to flow projection artifact, the true plexus boundaries between the superficial and deeper vasculature could not be well characterized, which led to segmentation at the INL/IPL border on SD-OCT (as shown in Figs 1 and 4). Unfortunately, this anatomic boundary yields unpredictable segmentation of the ICP between the superficial and deep slabs.

The PR-OCTA algorithm provides a new and more accurate way to investigate detailed retinal vascular anatomy in humans *in vivo*^18^. In this article, we used this new technology to advance retinal vascular imaging in several significant ways. First, we demonstrate that PR-OCTA can visualize four unique vascular plexuses in the human retina with distinct vascular patterns, which vary based on depth and location from the nerve. Secondly, using PR-OCTA one can visualize the inter-plexus spaces and the interconnecting vascular segments *in vivo*. Thirdly, PR-OCTA preserves *in situ* flow signal in deeper vessels, resulting in visualization of continuous capillary networks in the ICP and DCP. This was not possible with earlier attempts at projection suppression using slab-subtraction algorithms, which produced numerous gaps that disrupted the continuities of vascular networks deeper layers^16^,^21^,^25^. Finally, we were able to perform quantitative analysis of capillary density using PR-OCTA, and establish the relative and absolute troughs in vessel density that form natural boundaries between retinal vascular plexuses. These boundaries were defined in terms of retinal anatomic layers that could be easily segmented from structural OCT images. Based on what could be delineated on PR-OCTA, we propose a system of terminology for the retinal circulation (Figs 1, 4 and 5) that we hope will be useful for future clinical studies. These definitions are consistent with the detailed anatomy known from histologic studies^2^,^4^,^12^, but are more detailed than what was used in previous OCTA literature. This PR-OCTA vascular anatomic scheme is described below.

**Figure 2:**
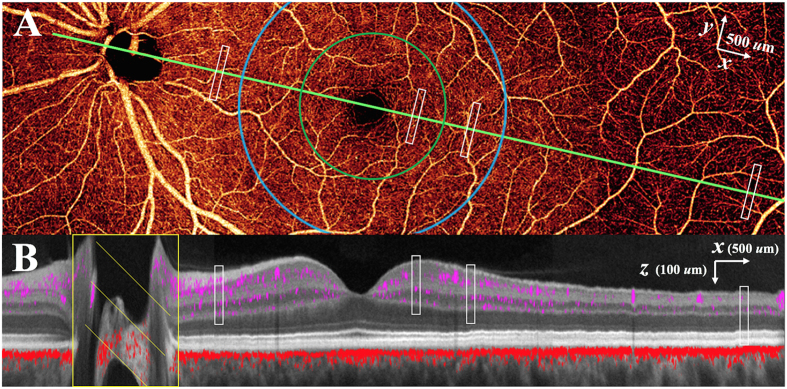
Projection-resolved optical coherence tomography angiography (PR-OCTA) in the left eye of a normal study participant. Four 4.5 × 4.5 mm OCTA volumes were montaged. (**A**) 14.2 × 4.5 mm *en face* OCTA of the inner retina. The cross-sectional image (**B**) is taken along the maculopapillary axis (green line joining the centers of the fovea and optic disc). (**B**) Color-composite cross-sectional OCTA (14.2 × 0.7 mm) showing retinal (purple) and choroidal (red) blood flow superimposed on gray scale reflectance image of static structures. The white rectangles in (**A**) and (**B**) represent the 0.1 × 0.8 × 0.25 mm (*x* × *y* × *z*) sampling regions at locations in the peripapillary, parafoveal (green circle), perifoveal (blue circle), and peripheral (7 mm temporal to fovea) retina for capillary density measurements.

The superficial vascular complex consists of the SVP and the RPCP. The SVP is a network of both large and small vessels connected directly to the retinal arteries and veins, and supplies all other vascular plexuses. In the macula, the SVP is arranged in a centripetal pattern that converges on the parafoveal capillary ring (Fig. 5). In the periphery, the superior and inferior circulations converge in an interleaved comb pattern similar to the retinal vasculature shown by FA^12^,^23^. Primate histology demonstrated that the SVP supplies all other vascular plexuses through vertical pre-capillary arterial segments, which typically ascend to the NFL (in the case of the RPCP) and descend to the deeper layers (ICP and DCP) off of the 1^st^ order branches from the main arcades and terminal extensions of the SVP arterial segments^2^,^4^. The post-capillary segments often drain directly into the main venous arcades^3^. We can now visualize these vascular networks using PR-OCTA including the interconnecting vessels and the laminar networks in the ICP and DCP (Fig. 6). The SVP vessel density decreases as a function of distance from the optic nerve (Fig. 7) as the surrounding GCL decreases in thickness (Fig. 2). These results correlate well with what is known from primate histology^2^.

The RPCP represents a unique capillary plexus in the peripapillary region with three defining characteristics from the original histology, which can be uniquely visualized within the SVC by segmenting on the NFL/GCL interface^4^,^5^. First, as opposed to the deeper SVP capillary plexus, the RPC travel along relatively longer and straighter paths than other plexuses (Fig. 5)^4^. Second, they have fewer anastomoses within the plane of the RPCP (Fig. 5) but are fed by pre-capillary arterial segments from the SVP and drained by post-capillary venous segments into the SVP. Third, though in these data we focused analysis on the maculopapillary axis, histology demonstrates that these vessels are located only in the posterior pole where they run alongside the dense NFL axons in an asymmetric butterfly distribution around the optic nerve and the vascular arcades^4^. Prior work has suggested that this layer nourishes the NFL, and therefore is densest in the peripapillary region and reduces in thickness as the NFL decreases, which is consistent with our findings (Figs 5 and 7)^2^,^4^,^5^.

As a whole, the SVC presents an extremely dense vascular network in the peripapillary region (Fig. 5) that decreases in density with distance from disc along the maculopapillary axis (Fig. 7). This agrees with previous histologic evidence – Snodderly *et al*. reported the percentage of retinal area covered by capillaries and found that it was highest in the peripapillary region, often exceeding 60%^2^.

The DVC consists of the ICP above the INL and the DCP below the INL. The capillaries of the ICP and DCP have a lobular configuration with no directional preference within their laminar planes. They consist of capillaries of uniform size (Fig. 5), with the exception of larger vessels that interconnect the plexuses (Fig. 6). From primate histology we know that these plexuses represent terminal anastomotic capillary networks supplied by vertically oriented interconnecting arteries and veins from the SVP^3^, which we can now demonstrate (Fig. 6). Confocal microscopy in *ex vivo* specimens has demonstrated that the ICP is situated among bipolar cell processes, and the DCP localizes to the region near the horizontal cells in the outer INL^26^, presumably due to local metabolic needs in the retina^3^. In addition, we demonstrated that these plexuses have relatively constant capillary density from the optic nerve to the periphery along the maculopapillary axis (Fig. 7), and merge approximately 6–7 mm temporal to the fovea, two novel findings that are facilitated by the wider field of view of OCTA compared to prior technologies^9^,^10^ and the improved *in situ* visualization of flow using PR-OCTA.

We have developed a system for segmenting the retinal circulation into 2 complexes and 4 plexuses using anatomic layers that could be delineated on structural OCT (Fig. 5). The appropriate segmentation scheme to be used for the display and quantitative analysis of *en face* PR-OCTA depends on the anatomic region. In the peripheral retina, the SVC and DVC are the 2 distinguishable layers. In the macula, the DVC can be divided into the ICP and the DCP to further improve the detection of capillary dropout and other vascular pathologies. In the peripapillary region, the SVC can be divided into the RPCP and the SVP. Around the foveal avascular zone (FAZ), the retinal plexuses converge to form a single parafoveal capillary loop and collectively define the borders of the FAZ. This is clearly visualized on PR-OCTA (Fig. 4) in agreement with early histological studies in primates^2^,^12^, which suggests that early reports highlighting FAZ area differences between superficial and deep plexuses may not be meaningful. Therefore, we recommend that the FAZ size be measured using an *en face* projection that include all retinal plexuses, rather than separating the FAZ area into superficial and deep FAZ areas, as has been done in early OCTA analyses^23^.

There are a number of limitations to this analysis. First, we report the values for only a small population of subjects. Further studies using PR-OCTA are needed to establish a normative database of retinal plexus that account for relevant demographic variable such as age, gender, race/ethnicity, and refractive error/axial eye length, in addition to biological variables such as oxygen saturation, caffeine use, and physical activity. Second, the analysis of PR-OCTA in diseased eyes are beyond the scope of this report. In some diseases, retinal anatomic boundaries may be difficult to identify and prevent detailed analysis of single plexuses.

The retina vascular complexes and plexuses that we have identified and measured with PR-OCTA have applications in retinal and optic nerve disease. In glaucoma^27^,^28^,^29^,^30^, and other neurodegenerative diseases^24^, structural OCT of the peripapillary NFL and the macular GCC have been helpful in diagnosis and monitoring. Atrophy and thinning of these structures are objective and precisely quantifiable disease indicators. However, retinal edema can confound thickness measurements, and in advanced glaucoma these structures are dominated by residual glial tissue and no longer serve as reliable disease indicators^31^. Optical coherence tomography angiography of the vascular supplies to these structure is a promising alternate approach to diagnose and monitor these diseases^6^,^32^,^33^,^34^,^35^. Using PR-OCTA, we have clearly identified the location of the RPCP in the peripapillary region, and the SVC in the macula. Accurate measurement of vascular perfusion in these plexuses could improve the assessment of glaucoma and other optic nerve diseases.

Using PR-OCTA to separate the retinal circulation into the SVC, ICP, and DCP in the macula, and SVC and DVC in the periphery, may be useful in the assessment of capillary dropout, microaneurysms, dilated shunt vessels, and other vascular pathologies associated with diabetic retinopathy^20^,^27^, and other ischemic diseases of the retina. There are also retinal diseases that are hypothesized to affect single plexuses, such as paracentral acute middle maculopathy^28^, and OCTA has demonstrated localized nonperfusion in this disease, even in the presence of a normal FA^29^,^30^. Using PR-OCTA to visualize capillary dropout in the ICP and DCP, as well abnormal vessels in all retinal layers, could be helpful in the assessment of these diseases. As FA transformed our understanding of retinal pathophysiology, OCTA may lead to an even deeper understanding and ultimately provide earlier and more specific identification of disease, and better outcomes for patients. By demonstrating the anatomic organization of the retinal vascular plexuses in normal human eyes, this work using PR-OCTA provides a rational schema for nomenclature and segmentation of the retinal vasculature in future OCTA publications.

## Materials and Methods

### Image Acquisition and Processing

Participants were recruited from the Casey Eye Institute. The research protocol was approved by the Oregon Health & Science University institutional review board and in compliance with the Declaration of Helsinki and HIPAA regulations. Informed consent was obtained from all human subjects. A commercial spectral-domain 70 kHz OCT instrument (RTVue-XR, Optovue) was used to obtain OCTA scans in healthy eyes. A commercial version of the split-spectrum amplitude-decorrelation angiography (SSADA) algorithm was used to detect blood flow^36^. The measurements were taken with the subject at rest in normal room air in a seated position. Blood pressure measurements were taken and confirmed to be normal. The axial resolution of the OCT system was 5 μm (full-width half-maximum) and the transverse beam spot diameter was 15 μm. Split-spectrum processing reduces the axial resolution to 20 μm for SSADA flow signal^36^. The axial voxel height was 3.1 μm and each axial (*z*) scan has 512 voxels. Each volumetric scan has 304 × 304 transverse points. Each set of volumetric image data was obtained by orthogonal registration and merging of 1 *x*-priority and 1 *y*-priority scans. The OCTA data from different participants were registered based on OCT structural information. Specifically, the x dimension (Fig. 2) was linearly scaled to register the disc and fovea center (from the middle of the optic disc to the middle of the fovea), each major structural layer (ILM + NFL, GCL + IPL, INL, OPL) was also scaled along z dimension (Fig. 2) to register the individual layers of all participants. The subject depicted in Fig. 2 image was used as the reference subject for x and z scaling for all other subjects, and was the youngest subject without refractive error. Thus, the variability in the x and z dimensions was minimized so that we could identify the anatomic location of the capillary plexuses as a function of the structural boundaries, rather than arbitrary depth measurements, which would vary by retinal thickness. The PR-OCTA algorithm identifies voxels with *in-situ* flow as those where reflectance-normalized decorrelation values are higher than all shallower voxels in the same axial scan line^25^ Fig. 8 provides a brief illustrated explanation of this algorithm. En face and cross-sectional angiograms were obtained by slab projection. Structural OCT images were obtained by averaging reflectance signal over the slab thickness^37^. Angiograms were obtained by maximum flow projection over the slab thickness.

### Depth-resolved capillary density calculation

**Figure 3:**
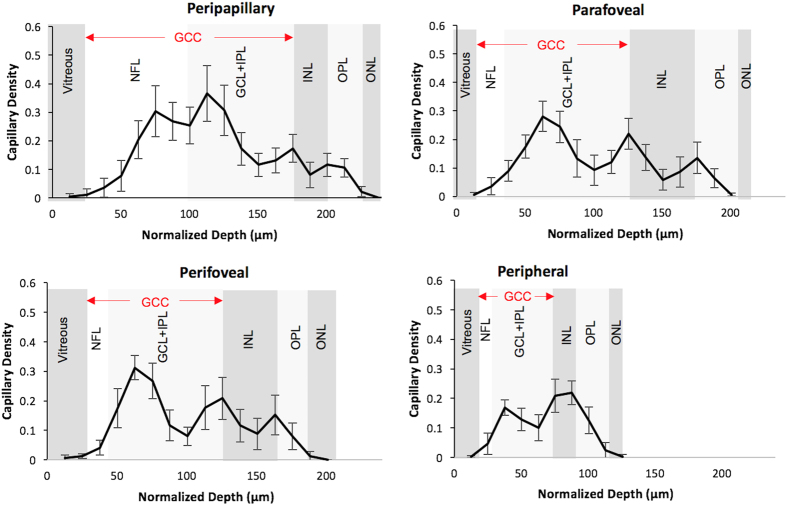
Retinal capillary density as a function of normalized depth and structural optical coherence tomography layers. Depth-resolved capillary density profiles are measured in representative areas (Fig. 2) in 4 anatomic regions. Population average and standard deviation capillary density measurements from the 9 normal human participants are shown. In all regions, a capillary density peak in GCL corresponds to superficial vascular plexus. In the peripapillary region, a peak within NFL corresponds to the radial peripapillary capillary plexus. In all regions except peripherally, a peak at inner border of INL corresponds to the intermediate capillary plexus (ICP) and a peak at outer border of INL corresponds to the deep capillary plexus (DCP). Peripherally, the ICP and DCP coalesce into one peak. The x dimension depth scale (corresponding to the z scale in Fig. 2) represents normalized depth with each labeled OCT structural layer normalized to the reference subject (shown in Fig. 2), minimizing the variability in this dimension but maintaining the anatomic relationship with structural OCT layers. (OCT = optical coherence tomography, NFL = nerve fiber layer, GCL = ganglion cell layer, IPL = inner plexiform layer, GCC = ganglion cell complex, INL = inner nuclear layer, OPL = outer plexiform layer plus Henle’s fiber layer, ONL = outer nuclear layer).

**Figure 4:**
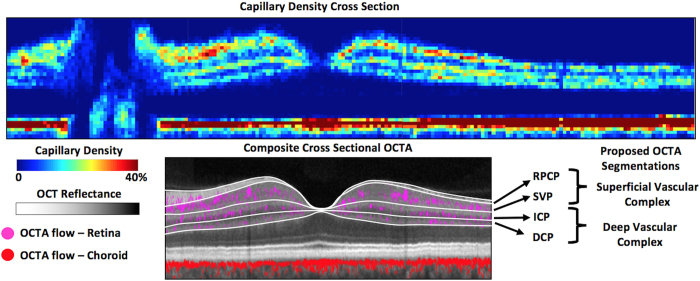
Cross-sectional capillary density map in the retina of a normal human participant, and proposed segmentation boundaries. The top 14.2 mm (*x*) by 0.7 mm (*z*) image was obtained by montaging four 4.5 × 4.5 mm PR-OCTA volumes and selecting a cross-sectional slab along the maculopapillary axis (Fig. 2). The capillary density is measured within super-voxels of 0.1 × 0.8 × 0.01 mm (*x* × *y* × *z*). Three layers of concentrated capillary density could be seen in the retina (top layers of upper image): superior vascular complex, intermediate capillary plexus, and deep capillary plexus. One layer of high capillary density is seen in the choriocapillaris (bottom layer). Proposed segmentation boundaries are showen in lower image (white lines) with corresponding structural OCT layers. (OCT = optical coherence tomography, RPCP = radial peripapillary capillary plexus, SVP = superficial vascular plexus, ICP = intermediate capillary plexus, DCP = deep capillary plexus, GCC = ganglion cell complex, OPL = outer plexiform layer, RPE = retinal pigment epithelium).

In order to visualize the depth of the retinal vascular plexuses (Fig. 3), the OCTA volumes were aligned at the RPE level. Then flow pixels at each depth (*z*) plane in 0.1 mm × 0.8 mm (*x* × *y*) mm sample areas were then counted and divided by the total number of pixels to yield a capillary density (units = percent flow per area). For the purposes of capillary density measurement in all plexuses, the areas occupied by larger vessels (arteries and veins) were excluded from the analysis, by filtering the signal using a signal intensity based thresholding algorithm to create a large vessel mask from the en face angiogram of the SVP (using a Gaussian window (20 × 20 pixels), then thresholding at a SSADA decorrelation value of 0.12) and keeping connected regions larger than 50 voxels. The same procedure was applied to the entire group. The purpose of this mask was to remove the measurement of the large vessels from estimates of “capillary density”. For the cross-sectional capillary density map (Fig. 4) each color-coded pixel in the cross section presents the capillary density in a super-voxel 0.1 mm wide (*x*) by 0.8 mm deep (*y*, perpendicular to the image plane) by 0.01 mm high (*z*) in units of % volume occupied by flow voxels within the super-voxel. The repeatability of this measurement for within-visit intra-subject repeatability was evaluated using the coefficient of variation.

### Segmentation boundaries for en face visualization of vascular plexuses

**Figure 5:**
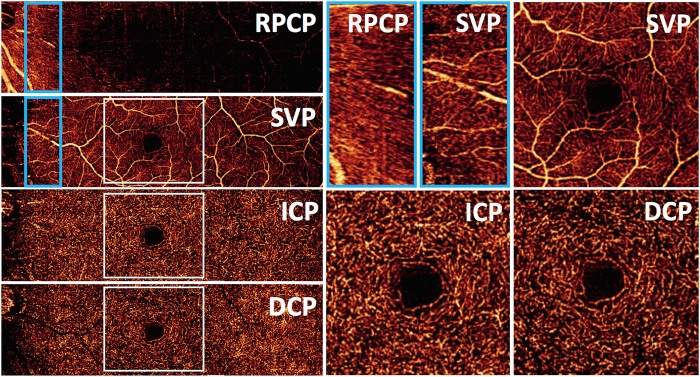
*En face* projection-resolved optical coherence tomography angiograms of four retinal vascular plexuses in the left eye of a normal human participant. The angiograms are formed by the montage of four 2 × 2 mm scans. The radial peripapillary capillary plexus (RPCP) is found in the nerve fiber layer (NFL) slab. The superficial vascular plexus (SVP) slab was predominantly located in the ganglion cell layer (GCL), and was segmented as the inner 80% of the ganglion cell complex (GCC, defined as the NFL + GCL + inner plexiform layer [IPL]), excluding the NFL. The intermediate capillary plexus (ICP) was segmented between the outer 20% of the GCC to the inner 50% of the inner nuclear layer (INL). The deep capillary plexus was segmented between the outer 50% of the INL and the outer plexiform layer (OPL). High magnification images of the peripapillary RPCP, and parafoveal vascular networks of the SVP, ICP, and DCP are presented at the right, from corresponding sections indicated with white squares.

**Figure 6:**
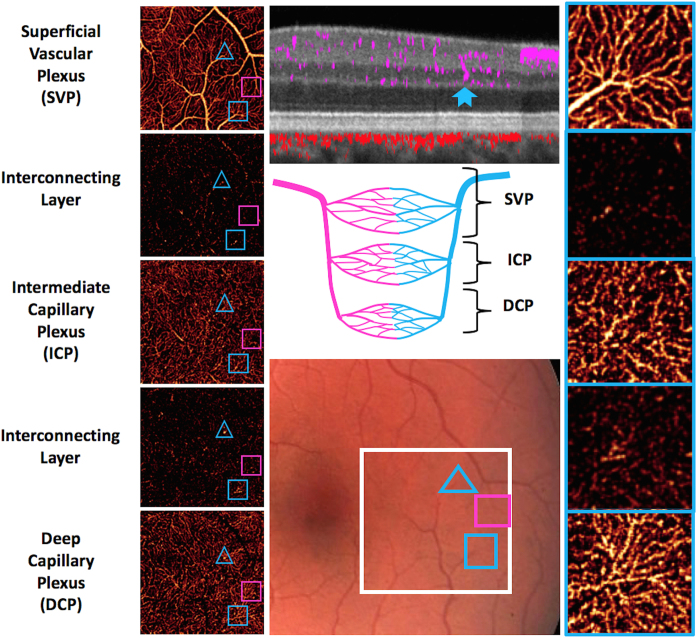
Retinal vascular plexuses and interconnecting layers in the macula. Color fundus photograph (middle bottom panel) demonstrates the 2 × 2 mm region of scan (white square). The *en face* PR-OCTA (left panels) are arrayed from the most superficial on top to the deepest at the bottom. The blue hollow triangles enclose a diving venule (identified on color fundus photograph) that can be seen in the cross-sectional PR-OCT (middle top panel) traversing from the SVP to the DCP (solid blue notched arrow). The hollow pink squares enclose a diving arteriole that can be seen in the SVP, ICP, DCP, and the interconnecting layer between the ICP and the DCP. The hollow blue squares enclose a diving venule that is clearly seen in all magnified *en face* PR-OCTA slabs (right panels). This venule gives rise to a radiating network of capillaries in all 3 plexuses. The cartoon (center panel) depicts the anatomical relationships between arterial and venous systems in the three vascular plexuses and the interconnecting layers.

**Figure 7:**
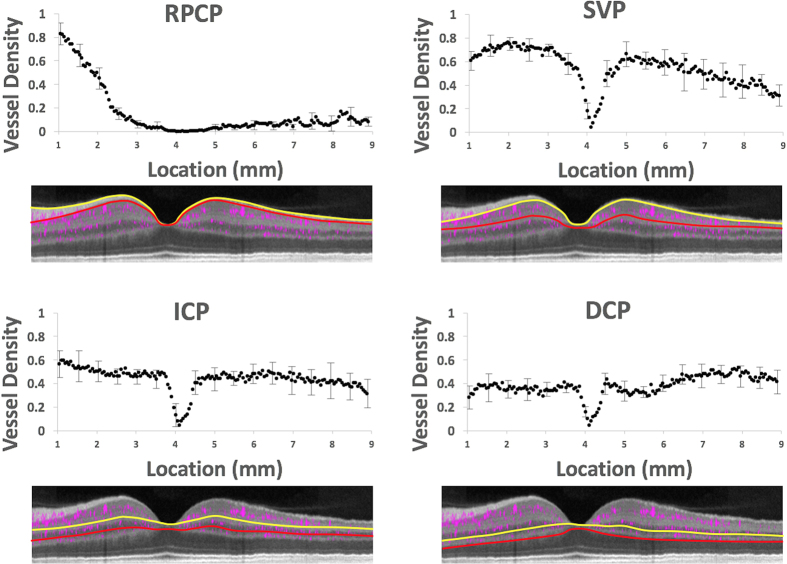
Transverse vessel density profiles in the 4 retinal vascular plexuses. Vessel density was calculated in *en face* projections of the plexuses along a 0.7 mm wide (y, perpendicular to image plane) swath, sampled every 0.05 mm (x) along the maculopapillary axis (Fig. 2). The locations are measured by distance from the edge of the optic disc. The yellow and red lines represent the superficial (yellow) and deep (red) segmentation boundaries for vessel density measurements for each plexus, which were averaged from 9 normal human participants and error bars representing standard deviation is shown every 10^th^ point. The x axis scale was normalized to a reference image for all participants at the disc and fovea. The transverse vessel density profile includes large vessels and is based on maximum flow projection across the full thickness of the plexuses (1–24 voxel thick slabs) and therefore have higher values than the depth-resolve capillary density profile (single-voxel thick slabs) shown before. (RPCP = radial peripapillary capillary plexus, SVP = superficial vascular plexus, ICP = intermediate capillary plexus, DCP = deep capillary plexus).

**Figure 8:**
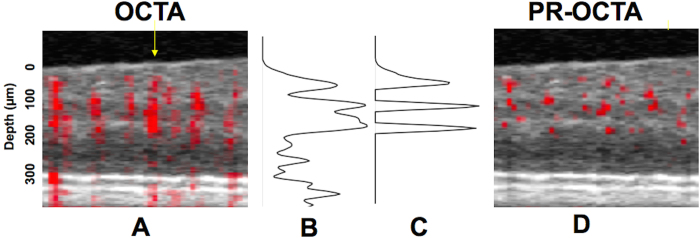
Illustration of the projection-resolved (PR) optical coherence tomography angiography (OCTA) algorithm. (**A**) and (**D**) Composite cross-sectional (B-scan) images before (**A**) and after (**D**) projection resolution. In these two images, suprathreshold decorrelation signal (red) is overlaid on the structural OCT (gray scale). The threshold distinguishes flow from background noise and is based on noise statistics. (**B**) Original axial profile of reflectance-normalized decorrelation signal (**C**) The projection resolution algorithm retains suprathreshold reflectance-normalized decorrelation signal that are higher than all voxels above (voxels classified as *in situ* flow in real vessels) and sets the remaining signal to zero (classified as flow projection artifacts).

Using data from the depth-resolved capillary density profile (Fig. 3), and comparing to the structural SD-OCT signal, we empirically defined segmentation boundaries that separated the vascular plexuses using structural SD-OCT boundaries. These boundaries (Fig. 5) were automatically processed (with manual verification), the same for all patients and were used throughout this article. The superficial vascular plexus (SVP) slab was defined as the inner 80% of the ganglion cell complex (GCC), which is commonly used for macular evaluation using SD-OCT (defined as the NFL + GCL + inner plexiform layer [IPL]). We then isolated the RPCP from the SVP by segmenting on the NFL/GCL interface. Thus, the SVC (SVP + RPCP) consisted of all vessels between the ILM and inner 80% of GCC. The intermediate capillary plexus (ICP) was segmented between the outer 20% of the GCC to the inner 50% of the inner nuclear layer (INL). The deep capillary plexus was segmented between the outer 50% of the INL and the outer plexiform layer (OPL). Thus, the DVC (ICP + DCP) is found in the anatomic layers consisting of the outer 20% of the GCC (outer IPL) to the OPL. In order to visualize the inter-plexus space, we created single voxel-thick *en face* OCTA images through the inter-plexus spaces in the IPL and INL (Fig. 6). The space between the SVP and ICP was found in the IPL, and the space between the ICP and the DCP was found in the INL.

### Data analysis

Statistical analysis was performed using Stata version 11.0 (College Station, TX). Peak vessel densities were compared using the t-test, and multivariate linear regression was performed with a significance of P < 0.05 being considered statistically significant.

## Additional Information

**How to cite this article**: Campbell, J. P. *et al*. Detailed Vascular Anatomy of the Human Retina by Projection-Resolved Optical Coherence Tomography Angiography. *Sci. Rep.* **7**, 42201; doi: 10.1038/srep42201 (2017).

**Publisher's note:** Springer Nature remains neutral with regard to jurisdictional claims in published maps and institutional affiliations.

## Figures and Tables

**Table 1:** Peak capillary density by plexus and anatomic location.

Location	Peripapillary	Parafoveal	Perifoveal	Peripheral
RPCP	0.30 + 0.09			
SVP	0.37 + 0.10	0.28 + 0.05	0.31 + 0.04	0.17 + 0.03
ICP	0.17 + 0.05	0.22 + 0.05	0.21 + 0.07	0.22 + 0.04
DCP	0.12 + 0.04	0.13 + 0.06	0.15 + 0.07	

Units are percent area occupied by flow pixels in 0.8´0.1 mm sampling areas shown in Fig. 2. The values shown are population mean ± standard deviation on 9 normal human participants.
